# Epigenetic deregulation of multiple *S100* gene family members by differential hypomethylation and hypermethylation events in medulloblastoma

**DOI:** 10.1038/sj.bjc.6603852

**Published:** 2007-06-19

**Authors:** J C Lindsey, M E Lusher, J A Anderton, R J Gilbertson, D W Ellison, S C Clifford

**Affiliations:** 1 Northern Institute for Cancer Research, Newcastle University, Newcastle upon Tyne, NE2 4HH, UK; 2 Department of Developmental Neurobiology, St Jude Children's Research Hospital, Memphis, TN 38105, USA

**Keywords:** medulloblastoma, S100, epigenetics, hypomethylation, hypermethylation

## Abstract

Deregulated expression of genes encoding members of the S100 family of calcium-binding proteins has been associated with the malignant progression of multiple tumour types. Using a pharmacological expression reactivation approach, we screened 16 *S100* genes for evidence of epigenetic regulation in medulloblastoma, the most common malignant brain tumour of childhood. Four family members (*S100A2*, *S100A4*, *S100A6* and *S100A10*) demonstrated evidence of upregulated expression in multiple medulloblastoma cell lines, following treatment with the DNA methyltransferase inhibitor, 5′-aza-2′-deoxycytidine. Subsequent analysis revealed methylation of critical CpG sites located within these four genes in an extended cell line panel. Assessment of these genes in the non-neoplastic cerebellum (from which medulloblastomas develop) revealed strong somatic methylation affecting *S100A2* and *S100A4*, whereas *S100A6* and *S100A10* were unmethylated. Assessed against these normal tissue-specific methylation states, *S100A6* and *S100A10* demonstrated tumour-specific hypermethylation in medulloblastoma primary tumours (5 out of 40 and 4 out of 35, respectively, both 12%) and cell lines (both 7 out of 9, 78%), which was associated with their transcriptional silencing. Moreover, *S100A6* hypermethylation was significantly associated with the aggressive large cell/anaplastic morphophenotype (*P*=*0.026*). In contrast, pro-metastatic *S100A4* displayed evidence of hypomethylation relative to the normal cerebellum in a significant proportion primary tumours (7 out of 41, 17%) and cell lines (3 out of 9, 33%), which was associated with its elevated expression. In summary, these data characterise complex patterns of somatic methylation affecting *S100* genes in the normal cerebellum and demonstrate their disruption causing epigenetic deregulation of multiple *S100* family members in medulloblastoma development. Epigenetic events affecting *S100* genes have potential clinical utility and merit further investigation as molecular biomarkers for this disease.

Epigenetic alterations play an important role in the development of medulloblastoma, an embryonal tumour of the cerebellum, which accounts for ∼20% of childhood brain tumours (Giangaspero *et al*, 2000; Lindsey *et al*, 2005). The S100 protein family is a large family of EF hand calcium-binding proteins of approximately 20 members (Donato, 2003; Marenholz *et al*, 2004), involved in the regulation of a variety of cellular processes including cell growth and cell cycle regulation, differentiation, transcription and motility. Sixteen members of the gene family (*S100A1*–*S100A16*) map within a 1.65 Mb region at chromosome 1q21.3 (Figure 1A) (Marenholz *et al*, 2004).

Members of the *S100* gene family show divergent patterns of cell and tissue-specific expression, and the expression of specific family members is disrupted in a range of diseases including cancer (Heizmann *et al*, 2002; Marenholz *et al*, 2004; Heizmann, 2005). Overexpression of several of the S100 proteins is thought to be involved in tumour progression, such as S100A4, which is upregulated in many cancers including medulloblastoma and promotes angiogenesis and metastasis (Hernan *et al*, 2003; Emberley *et al*, 2004; Helfman *et al*, 2005; Garrett *et al*, 2006) and S100P which is associated with metastasis in breast and pancreatic cancer (Arumugam *et al*, 2005; Wang *et al*, 2006). Other S100 proteins are downregulated in tumours and have putative tumour suppressor roles, including S100A2 and S100A6 in prostate cancer (Rehman *et al*, 2005).

Expression of several members of the *S100* family, including *S100A2, S100A4, S100A6* and *S100P*, is known to be regulated epigenetically, by methylation of key CpG sites within the genes or their promoters (Wicki *et al*, 1997; Rosty *et al*, 2002; Sato *et al*, 2004; Rehman *et al*, 2005). This regulation has been postulated to be important in controlling the cell type-specific expression of *S100* genes, as methylation associated with transcriptional silencing of these genes in normal somatic tissues has been found to occur in a tissue-specific manner (Lesniak *et al*, 2000; Rosty *et al*, 2002; Sato *et al*, 2004). Accordingly, the normal regulation of individual *S100* family members can be disrupted during tumourigenesis, by aberrant gene-specific methylation events, which have been demonstrated in diverse cancer types including lung, prostate, pancreatic and colon cancer (Wicki *et al*, 1997; Nakamura and Takenaga, 1998; Lesniak *et al*, 2000; Feng *et al*, 2001; Rosty *et al*, 2002; Rehman *et al*, 2004, 2005; Sato *et al*, 2004). However, the epigenetic status of *S100* genes in medulloblastomas and the normal cerebellum has not been previously investigated.

In this study, we examined 16 members of the *S100* gene family for evidence of methylation-dependent epigenetic regulation in medulloblastoma, using a pharmacological expression reactivation approach, involving microarray analysis of gene expression changes induced by the treatment of medulloblastoma cell lines with inhibitors of DNA methylation. Four gene family members, *S100A2, S100A4, S100A6* and *S100A10*, displayed consistent and confirmed evidence of methylation-dependent upregulation following treatment. The methylation status of these genes was therefore investigated in detail in medulloblastoma primary tumours, cell lines and the normal cerebellum, to identify and characterise any epigenetic events affecting them, and to assess any potential biological roles and/or clinicopathological significance in medulloblastoma pathogenesis.

## MATERIALS AND METHODS

### Cell lines

Nine independently derived medulloblastoma cell lines (DAOY, D283 Med, MHH-MED-1, MEB-MED8A, D341 Med, D384 Med, D425 Med, D556 Med and UW228-3) were studied. All cells were grown under recommended culture conditions provided by suppliers (see acknowledgements) and cell line identity was confirmed before use by karyotyping (data not shown). Cell line DNA was extracted using the Qiagen DNeasy kit (Qiagen, Crawley, UK).

### Primary tumours and normal control samples

A cohort of 41 primary medulloblastomas was analysed including representatives of all the major histopathological subtypes (25 classic, six large cell/anaplastic and 10 nodular/desmoplastic)(Giangaspero *et al*, 2000). Patients (15 females and 26 males) ranged in age from 1.3 to 19 years (seven infants <3 years, 30 children 3–16 years and four adults >16 years). Six cerebellar samples were analysed consisting of post-mortem material from three fetuses (18- 19- and 22-weeks gestational age), one infant (newborn) and two adults (60 and 67 years) who had died of non-neoplastic conditions. DNA was extracted from frozen tissues using standard methods and from formalin-fixed, paraffin-embedded tissue using a Nucleon hard tissue kit (Amersham Biosciences, Little Chalfont, UK). Local Ethical Committee and Institutional Review Board approval was obtained for the collection, storage and biological study of all material.

### 5′-Aza-2′-deoxycytidine (5-aza CdR) treatment

Four cell lines (DAOY, D283 Med, D425 Med and MEB-MED8A) were grown in the presence or absence of the demethylating agent 5-aza CdR (5 *μ*M) (Sigma-Aldrich, Poole, UK) for 3 days. Medium was renewed daily.

### Microarray analysis

Total RNA was extracted from cell lines D283 Med, D425 Med and MEB-MED8A, which had been grown in parallel cultures with or without 5-aza CdR treatment. These cell lines were selected for analysis as they exhibit characteristic chromosomal defects representative of primary medulloblastomas (Langdon *et al*, 2006). RNA was extracted from 10^7^ cells using Trizol reagent (Invitrogen, Paisley, UK) according to manufacturer's instructions. Microarray expression analysis was performed at the Newcastle University microarray facility; RNA was converted into biotin-labelled cRNA and hybridised to the Human U133A array according to manufacturer's protocols (Affymetrix, Santa Clara, CA, USA). MAS5 software (Affymetrix) was used for data processing, normalisation and calculation of signal intensities. Signal intensities were compared between treatment conditions using Microsoft Excel software (Microsoft, Reading, UK).

### Analysis of *S100* gene methylation status

Bisulphite treatment of DNA was carried out using a CpG genome DNA modification kit (Serologicals, Livingston, UK) according to the manufacturer's instructions. The promoter and exon1 regions of *S100A2* and *S100A6* and a region within the first intron of *S100A4* were amplified following bisulphite treatment using previously published primers and conditions (Rosty *et al*, 2002; Rehman *et al*, 2005). The promoter region of *S100A10* (Huang *et al*, 2003) was analysed using primers designed by ‘MethPrimer’ (Li and Dahiya, 2002) to amplify a region of the CpG island 400–652 bp relative to the transcriptional start site. Primer sequences were S100A10F 5′-ATTATTTGTTGGATGATTTTGTAGG-3′ and S100A10R 5′-ACACAAAAAAATAAATCCCCTATTC-3′ (designed to amplify the antisense strand following bisulphite conversion). Thirty nanograms of bisulphite treated DNA was used per reaction. PCR products were amplified using standard conditions with an annealing temperature of 60°C. PCR products were directly sequenced with a CEQ DTCS kit (Beckman Coulter, High Wycombe, UK). Sequenced products were analysed on a CEQ 2000XL DNA analysis system (Beckman Coulter), and the methylation status at each CpG residue determined by assessment of the relative peak intensities.

Combined bisulphite and restriction analysis (COBRA (Xiong and Laird, 1997) of *S100A4* was carried out by overnight digestion of the 142 bp PCR product at 37°C with the *HypCH4IV* restriction endonuclease (New England Biolabs, Hitchin, UK), which has the recognition sequence 5′-ACGT-3′ and which cuts twice within the product if fully methylated before bisulphite conversion to give 100, 26 and 16 bp fragments. Digested PCR products were separated on a 4% Nusieve 3 : 1 agarose gel in 1 × TBE (0.09 M Tris-Borate, 0.002 M EDTA pH 9) and stained with ethidium bromide, before visualisation by transillumination under ultraviolet (UV) light. The unmethylated control for COBRA and bisulphite sequence analysis consisted of a pool of 20 newborn cord blood DNAs, the methylated control was universal methylated DNA (Serologicals Corporation, Livingston, UK). Representative examples of PCR products showing different digestion patterns were sequenced as described above, to determine relative peak intensities.

### Reverse transcription PCR (RT–PCR)

RNA was extracted from 10^7^ subconfluent cells using Trizol reagent (Invitrogen, Paisley, UK) according to manufacturer's instructions. One microgram of total RNA was used to synthesise cDNA using a reverse transcription system (Promega, Southampton, UK). Equivalent amounts of this cDNA were used for PCR amplification of the respective *S100* gene, *RASSF1A* and *ACTB* transcripts. RT–PCRs for *S100A2, S100A4* and *S100A6* were carried out using previously published primers and conditions (Rosty *et al*, 2002; Rehman *et al*, 2005). RT primers for *S100A10* were designed using transcript information obtained from the Ensembl genome browser (www.ensembl.org; Gene ID, ENSG00000197747). Primer sequences were S100A10rtF (in exon 2) 5′-TTCACAAATTCGCTGGGGATAA-3′ and S100A10rtR (in exon 3) 5′-AACTGCTCATTTCTGCCTACTTCT, which amplified a 264 bp product. PCR products were amplified using standard conditions with an annealing temperature of 54°C. RT–PCR of *RASSF1A* was used as a positive control and was carried out according to procedures published previously (Lusher *et al*, 2002). RT–PCR of *ACTB* (encoding *β*-actin, a housekeeping gene) was used as a control for RNA concentration and integrity and was carried out using previously published primers and conditions (Horikoshi *et al*, 1992). PCR products were electrophoresed on a 2% agarose gel in 1 × TBE and stained with ethidium bromide, before visualisation by transillumination under UV light.

## RESULTS

### Re-expression of *S100* family members following demethylating treatment of medulloblastoma cell lines: identification of candidate epigenetically regulated genes

**Figure 1:**
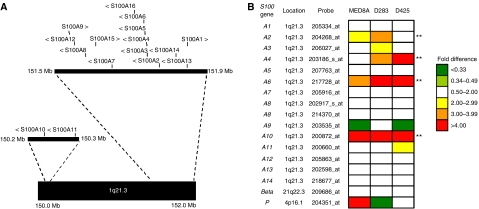
Methylation-dependent regulation of *S100* genes in medulloblastoma. (**A**) The *S100* gene cluster at 1q21.3, showing the position (vertical lines) and direction of transcription (>, <) of each gene. Physical positions are based on NCBI Assembly 36 of the human genome (see http://www.ncbi.nlm.nih.gov/). Genes not belonging to the *S100* family have been omitted for clarity. (**B**) Methylation-dependent changes in expression of 16 *S100* genes in three medulloblastoma cell lines (MEB-MED8A, D283Med, D425Med), following treatment with the DNA methyltransferase inhibitor, 5-aza-CdR (5 *μ*M, 72 h), determined by analysis using the Affymetrix U133A Human expression array (sequence identities of probes indicated were verified independently). Fold-changes in expression detected by each probe are shown, based on signal intensities assessed by MAS5 software (Affymetrix). Four genes (marked^**^) displayed evidence of positive methylation-dependent regulation in ⩾2 cell lines; *S100A2*, *S100A4*, *S100A6* and *S100A10*.

To identify *S100* genes showing evidence of methylation-dependent transcriptional regulation in medulloblastoma, three medulloblastoma cell lines (D425Med, D283Med and MEB-MED8A) were cultured in the presence or absence of the demethylating agent, 5-aza CdR. Resultant expression changes were assessed using the Affymetrix Human U133A array. The U133A array contains probe sets which recognise 16 *S100* genes (*S100A1-S100A14, S100β* and *S100P*), for which probe sequence identity and specificity could be verified using the BLAST sequence alignment tool (http://www.ncbi.nlm.nih.gov/BLAST/). Changes in probe signal intensity following 5-aza CdR treatment were calculated and genes showing an expression increase >2-fold were classed as being significantly upregulated, and thus showing evidence of methylation-dependent transcriptional regulation. Four independent S100 transcripts showed evidence of transcriptional upregulation in multiple cell lines (Figure 1B); *S100A6* and *S100A10* were upregulated in all three cell lines, while *S100A2* and *S100A4* were upregulated in two cell lines. In addition, *S100A3, S100A11* and *S100P* were each upregulated in one cell line, and the remaining *S100* genes were not significantly upregulated in any cell line.

**Figure 2:**
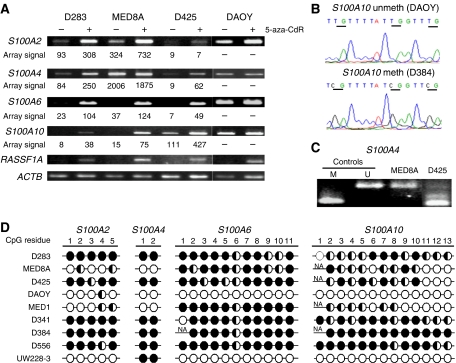
Epigenetic inactivation of *S100* genes by promoter hypermethylation in medulloblastoma cell lines. (**A**) Expression analysis of *S100* genes in medulloblastoma cell lines. RT–PCR analysis of *S100A2*, *S100A4, S100A6* and *S100A10* is shown for cell lines grown in the presence (+) or absence (−) of the demethylating agent 5-aza CdR (5 *μ*M, 72 h). Probe signal intensities generated for each gene in independent microarray experiments (see Figure 1A) are also given for comparison. *RASSF1A* (positive methylation-regulated gene) and *ACTB* (housekeeping) controls are also shown. (**B**) Representative examples of bisulphite sequence analysis. Two electropherograms showing analysis of three CpG sites within the *S100A10* promoter from a highly methylated cell line (D384) and an unmethylated cell line (DAOY). Individual CpG sites are underlined. (**C**) Representative examples of COBRA analysis of *S100A4*. PCR products from bisulphite-treated cell line DNA were digested with *HpyCH4IV*, which cuts the product twice when CpG sites are methylated, generating products of 100 bp (shown) and 26 and 16 bp (not shown). Unmethylated control (U) was normal blood DNA, methylated control (M) was universal methylated DNA (Serologicals Corporation, Livingston, UK). (**D**) Methylation status analysis of CpG residues within the promoter-associated CpG islands of *S100A2, S100A6* and *S100A*10 and first intron of *S100A4* in nine medulloblastoma cell lines, determined by bisulphite sequencing and estimation of relative peak heights. *S100A4* PCR products representative of different digestion patterns were sequenced and used to assign methylation levels to samples showing equivalent digestion patterns. Filled circles, >75% methylation; half-filled circles, 25–75% methylation; empty circles, <25% methylation; NA, not analysed.

The expression changes observed for *S100A6, S100A10, S100A2* and *S100A4* on array analysis were next validated in an independent series of experiments by RT–PCR (Figure 2A). The increases in expression observed by microarray for these genes following 5-aza CdR treatment were also clearly seen using RT–PCR methods, with good concordance between results obtained using the two techniques (Figure 2A). Reverse transcription–PCR analysis following demethylation treatment was also extended to a fourth medulloblastoma cell line, DAOY, which did not appear to show altered expression of any of the four *S100* genes examined following 5-aza CdR treatment, despite clear evidence of re-expression of the *RASSF1A* control transcript.

### The methylation status of *S100A2*, *S100A4*, *S100A6* and *S100A10* in medulloblastoma cell lines is associated with methylation-dependent transcriptional silencing

*S100* genes with confirmed evidence of methylation-dependent upregulation in ⩾2 medulloblastoma cell lines were selected for direct analysis of their DNA methylation status. The methylation status of CpG sites within the promoter/exon 1 regions of *S100A6* and *S100A2*, which have previously been identified as being involved in their epigenetic regulation (Rehman *et al*, 2005), was established in a panel of nine medulloblastoma cell lines by bisulphite sequencing. Both genes were frequently methylated (in 7 out of 9 and 8 out of 9 lines, respectively). *S100A6* displayed extensive methylation in D283 Med, D425 Med, MEB-MED8A, MHH-MED-1, D341 Med, D384 Med and D556 Med; the methylation of *S100A6* in D283 Med, D425 Med, MEB-MED8A is in accordance with its observed methylation-dependent transcriptional silencing and re-activation by 5-aza CdR in these cell lines. In contrast, DAOY and UW228-3 were completely unmethylated, correlating with the higher level of expression and the lack of expression change following demethylating treatment, seen in DAOY (Figure 2A and D). *S100A2* showed more variable methylation patterns, with methylation ranging from partial methylation at one site (DAOY) to complete methylation at all sites (D283 Med) (Figure 2D). However, the observed methylation patterns again correlated well with methylation-dependent transcriptional silencing (Figure 2A and D), except in the case of D425 Med, which is densely methylated and transcriptionally silenced, but does not undergo significant re-expression following 5 aza CdR treatment, suggesting that methylation-independent mechanisms are involved in its silencing.

Methylation analysis of *S100A10* has not been performed previously; however, the promoter and associated CpG island have been characterised. (Huang *et al*, 2003) Primers were designed using MethPrimer (Li and Dahiya, 2002) to assess the methylation status of a region of the *S100A10* promoter-associated CpG island (400–652 bp relative to the transcriptional start) site by bisulphite sequencing (see Figure 2B and D). This region was methylated at multiple CpG residues in 7 out of 9 cell lines, including all three cell lines, which showed evidence of transcriptional silencing and methylation-dependent upregulation of *S100A10* following treatment with 5-aza CdR (D283 Med, D425 Med, MEB-MED8A), but was not methylated in DAOY, which showed higher endogenous expression levels and no clear change in expression following demethylating treatment (Figure 2A), indicating that methylation of this region is involved in the transcriptional silencing and epigenetic regulation of *S100A10*.

*S100A4* does not contain a promoter-associated CpG island, but has been shown to be epigenetically regulated by methylation of critical intragenic CpG sites (Rosty *et al*, 2002). We therefore assessed the methylation status of two CpG sites within its first intron, whose methylation has been previously correlated with expression (Rosty *et al*, 2002). Methylation was assessed by COBRA (Figure 1C), with representative examples (*n*=3) of each digestion pattern confirmed by bisulphite sequencing. These two CpG sites were fully methylated in 6 out of 9 cell lines, and their methylation status correlated well with the expression patterns observed; reduced expression and 5-aza CdR-associated upregulation was seen in methylated cell lines (D283 Med, D425 Med), but not in unmethylated cell lines (MEB-MED8A, DAOY) (Figure 1; Figure 2A and D). These data were reinforced by extension of RT–PCR expression analysis of *S100A4* to two further cell lines (MHH-MED1, D556 Med), which produced the anticipated expression patterns; unmethylated MHH-MED1 showed higher endogenous expression and no evidence of methylation-dependent regulation, whereas the methylated D556 Med cell line displayed transcriptional silencing and methylation-dependent upregulation following 5-aza CdR treatment (data not shown).

### Methylation status of *S100* genes in the normal cerebellum

**Figure 3:**
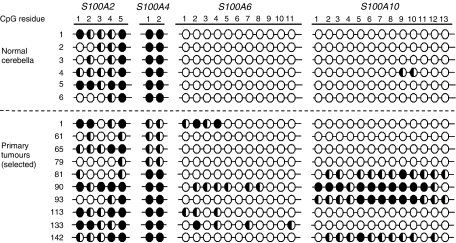
Methylation status of *S100* genes in primary medulloblastomas and the normal cerebellum. Analysis of methylation at CpG residues within the promoter-associated CpG islands of *S100A2*, *S100A6* and *S100A10* and the first intron of *S100A4*, is shown in normal cerebella and selected medulloblastoma primary tumours, determined by bisulphite sequencing and estimation of relative peak heights (as described in Figure 2). Filled circles, >75% methylation; half-filled circles, 25–75% methylation; empty circles, <25% methylation.

Gene-specific methylation can be a feature of normal somatic tissues (Strathdee *et al*, 2004). It is therefore crucial to establish the methylation status of candidate genes in normal non-neoplastic tissues before any role for aberrant methylation patterns in tumourigenesis can be assessed. Six normal cerebellar samples, comprising fetal, infant and adult examples were therefore assessed for the methylation status of all four *S100* genes under investigation (Figure 3). For *S100A4*, *S100A6* and *S100A10*, consistent methylation patterns were observed across all samples. *S100A6* and *S100A10* were somatically unmethylated, with no evidence of methylation of either promoter-associated CpG island observed, with the exception of one sample which showed minimal methylation (<25%) at 2 out of 13 sites in the *S100A10* CpG island. In contrast, *S100A4* was somatically methylated, showing evidence of high-level methylation at both CpG residues assessed in all normal cerebellar samples. *S100A2* also showed evidence of methylation in the normal cerebellum, although the methylation patterns detected were variable between samples, both in terms of the number of methylated CpG residues and the extent of methylation observed at these residues.

### Aberrant methylation patterns identify roles for *S100* genes in medulloblastoma pathogenesis

*S100* gene methylation patterns were next investigated in a series of primary medulloblastomas, and, together with patterns previously observed for medulloblastoma cell lines (Figure 2), were assessed against the normal methylation patterns observed for each gene in the cerebellum, for evidence of altered methylation states. In the majority of cases, methylation patterns observed in medulloblastomas corresponded to the methylation status of the normal cerebellum (Figure 3). For *S100A6* and *S100A10*, the majority of medulloblastomas were unmethylated (35 out of 40 and 31 out of 35 of analysable samples, 87.5 and 88.5% respectively); however five tumours (12.5%) and seven cell lines (78%) showed evidence of hypermethylation of the *S100A6* promoter relative to the normal cerebellum. *S100A10* was hypermethylated in four tumours (11.4%) and seven cell lines (78%), relative to the normal cerebellum. Direct analysis of expression in tumours was not possible, as RNA was not available for the majority of the cohort; however, the hypermethylation of *S100A10* observed in primary tumours is of a comparable level to that seen in the cell lines, consistent with its epigenetic transcriptional inactivation and candidate tumour suppressor role in disease pathogenesis (Figures 2A, D and 3). The hypermethylation observed for *S100A6* was less pronounced in primary tumours (affecting a limited number of CpG residues) than cell lines and further work is needed to determine whether this level of methylation leads to transcriptional silencing.

In contrast, all medulloblastomas showed some evidence of methylation of the CpG regions assessed in *S100A4* and *S100A2*. Whereas the normal cerebellum and the majority of primary medulloblastomas (34 out of 41) and medulloblastoma cell lines (6 out of 9) demonstrated complete methylation (>75%) of the *S100A4* intronic CpG sites analysed, a subset of primary tumours (17%; 7 out of 41) and cell lines (33%; 3 out of 9) were undermethylated in comparison, displaying either partial methylation or no evidence of methylation at each site (Figures 2D and 3). Considered alongside the methylation-dependent transcriptional silencing observed for *S100A4* in medulloblastoma cell lines (Figure 2A), these findings support (i) the somatic hypermethylation-associated transcriptional silencing of *S100A4* in the normal cerebellum, and (ii) tumour-specific hypomethylation of *S100A4* in a subset of medulloblastomas, consistent with elevated expression and a pro-tumourigenic role.

Patterns of methylation observed for *S100A2* were complex; evidence of methylation was observed in all primary tumours tested; however, considerable variation was seen in both the number of CpG residues affected and the extent of methylation observed at individual CpG residues (Figure 3). A comparable incidence and variability of patterns of methylation were also detected in medulloblastoma cell lines and the normal cerebellum, and our data do not therefore provide any clear evidence to indicate the existence of aberrant tumour-specific patterns of *S100A2* methylation in medulloblastomas (Figures 2D and 3). In view of the methylation-dependent transcriptional silencing associated with a range of *S100A2* methylation states in medulloblastoma cell lines (Figure 2A and D), our data suggest that *S100A2* methylation in the normal cerebellum is associated with its somatic epigenetic transcriptional silencing, but more detailed investigations are now required to explore any role for the epigenetic deregulation of *S100A2* in medulloblastoma.

### Clinicopathological significance of *S100* methylation events in medulloblastomas

Tumour-specific methylation patterns detected for the *S100A4, S100A6* and *S100A10* genes were next assessed against basic clinical and pathological information available for all tumours (age, sex, histopathological subtype), to make a preliminary assessment of any clinicopathological significance in disease development. *S100A6* methylation showed a statistically significant association with the large cell/anaplastic histopathological morpho-phenotype (50%; (3 out of 6) of large cell/anaplastic tumours *vs* 8% (2 out of 25) of classic and 0% (0 out of 9) of nodular/desmoplastic tumours; *P*=0.026 by Fisher's exact test). No further significant associations were found for any gene.

## DISCUSSION

This study demonstrates a role for the epigenetic deregulation of three members of the *S100* gene family, *S100A4, S100A6* and *S100A10*, in medulloblastoma tumourigenesis. Furthermore, our data provide evidence for the control of *S100* gene expression in the normal cerebellum by gene-specific somatic methylation events, and highlight the importance of assessing epigenetic events in tumour development in the context of normal tissue-specific methylation patterns.

Epigenetic deregulation of gene expression plays an important role in the development of medulloblastoma, and studies of DNA methylation have identified a series of putative tumour suppressor genes for this disease; however, analysis has so far been restricted to a limited number of candidate genes (Lindsey *et al*, 2005). In this study, we therefore used a pharmacological expression reactivation approach, to allow a more global analysis of gene expression changes using expression microarrays. Using this analysis to assess members of the *S100* gene family for evidence of methylation-dependent regulation, we have successfully identified four *S100* family members which showed upregulation in multiple cell lines following demethylation treatment, and were subsequently shown to be associated with specific DNA methylation events. Our findings thus strongly support the value of this experimental approach for the identification of novel methylated genes relevant to both normal development and oncogenesis, and provide important precedent for the epigenetic deregulation of *S100* gene expression in medulloblastoma. Moreover, the role of methylation events in the regulation of *S100* gene expression in the normal and malignant cerebellum may yet be more extensive than recognised currently. Our investigations of *S100* gene DNA methylation status were limited to four genes that displayed evidence of methylation-dependent regulation in multiple cell lines. Additional *S100* genes either (i) showed evidence of methylation-dependent regulation in a more limited number of cell lines (<2; *S100A3, S100A11, S100P* and *S100G*), and were therefore not investigated further in the present study, or (ii) were not represented on the U133A expression microarray (e.g., *S100A15*), suggesting that more systematic investigations are now required to investigate any roles for DNA methylation events in the epigenetic regulation of further *S100* family members in medulloblastoma.

Two of the genes identified by this approach, *S100A6* and *S100A10*, were each hypermethylated in a proportion of both medulloblastoma cell lines and primary tumours (each in ∼12% of cases), but not in the normal cerebellum. The higher frequency of hypermethylation observed for both genes in cell lines than tumours may reflect either a limited representation of the clinical and pathological diversity of primary medulloblastomas by these cell lines (Langdon *et al*, 2006), or an involvement of culture-related *de novo* methylation events (Jones *et al*, 1990; Pantoja *et al*, 2005). Nonetheless, for both genes a significant frequency of tumour-specific methylation was observed in primary tumours, which was associated with transcriptional silencing in medulloblastoma cell lines, indicating epigenetic inactivation and candidate tumour suppressor roles for *S100A6* and *S100A10* in primary medulloblastoma development. *S100A6* has been shown to be expressed in a cell type-specific manner in the brain, with expression present in subsets of neurons including granule cells of the cerebellum (Filipek *et al*, 1993). Consistent with this observation, the *S100A6* promoter was unmethylated in the normal cerebellum in the present study. In other tumour types, *S100A6* has also been reported to be hypermethylated in prostate cancer (Rehman *et al*, 2005). *S100A10* is also expressed in the brain (Zimmer *et al*, 2005). To our knowledge, the present study is the first to demonstrate that *S100A10* expression can be deregulated by methylation of its promoter-associated CpG island in tumourigenesis. The finding that *S100A10* was hypermethylated in medulloblastoma cell lines and tumours was unexpected, as its overexpression has previously been associated with plasminogen-dependent cellular invasiveness in fibrosarcoma and colorectal cancer cells (Choi *et al*, 2003; Zhang *et al*, 2004). However, *S100A10* can interact with a variety of proteins and is involved in diverse processes (reviewed in Santamaria-Kisiel *et al*, 2006), and studies to clarify its role in medulloblastoma tumourigenesis are now required.

In contrast, we have demonstrated consistent evidence of methylation of *S100A2* and *S100A4* in the normal cerebellum. The methylation patterns observed for both genes are comparable to those associated with transcriptional silencing in medulloblastoma cell lines, suggesting that expression of these genes is reduced or silenced epigenetically in the normal non-neoplastic cerebellum. There is growing evidence that a number of genes are epigenetically silenced by somatic methylation in normal tissues (reviewed in Strathdee *et al*, 2004). Previously we have shown evidence of partial methylation of *CASP8, HIC1* and *EDNRB* (Lindsey *et al*, 2004) in non-neoplastic cerebella. As less than 50 genes have been analysed for methylation in medulloblastoma and the normal cerebellum (Lindsey *et al*, 2005); this raises the possibility that somatic methylation in the normal cerebellum may be a feature of a significant number of genes, although for some it may be restricted to specific cell types within the cerebellum. In addition to cell type-specific methylation (Lesniak *et al*, 2000; Rosty *et al*, 2002; Sato *et al*, 2004), members of the *S100* gene family have been shown to undergo temporal methylation changes during development, for example *S100β*, becomes demethylated during astrocyte development in the mouse brain correlating with the time when its expression commences at E14 (Namihira *et al*, 2004). This raises the possibility that aberrant methylation in embryonal tumours such as medulloblastoma may reflect a failure of developmentally regulated methylation changes. The identification of normal tissue-specific methylation in the non-neoplastic cerebellum emphasizes the importance of including relevant somatic tissue controls in medulloblastoma methylation studies (Lindsey *et al*, 2004; Lindsey *et al*, 2005).

*S100A4* showed complete methylation of the intronic CpG sites analysed in all normal cerebellar samples, consistent with its epigenetic transcriptional silencing (see above). *S100A4* has been previously shown to be methylated in other normal tissues, including pancreatic tissue (Rosty *et al*, 2002). Three medulloblastoma cell lines were unmethylated for *S100A4*, and were associated with higher gene expression unaffected by demethylation treatment (in contrast to methylated lines, which were associated with methylation-dependent transcriptional silencing), suggesting that they had undergone selection for hypomethylation and overexpression of *S100A4* during the tumourigenic process. No tumour samples were completely unmethylated at *S100A4*; however, 7 out of 41 (17%) of tumours displayed reduced levels of methylation compared to the normal cerebellum. *S100A4* hypomethylation is also a feature of other cancers, including pancreatic and colon adenocarcinoma (Nakamura and Takenaga, 1998; Rosty *et al*, 2002). *S100A4* promotes angiogenesis and extracellular matrix degradation through its upregulation of specific matrix metalloproteases (reviewed in Helfman *et al*, 2005) and high levels of *S100A4* expression are associated with metastatic progression in a wide range of cancers including medulloblastoma (Hernan *et al*, 2003). Current findings are therefore consistent with a role for the oncogenic activation of *S100A4* by hypomethylation-associated expression upregulation in medulloblastoma tumourigenesis.

*S100A2* has been postulated in certain studies to play a suppressor role in tumour development, as it shows reduced expression in a range of cancer types, while other investigators have been unable to show any clear difference in methylation patterns between tumours and normal tissue (Rehman *et al*, 2005). *S100A2* methylation was detected in all normal cerebella, cell line and tumour samples tested; however, significant variability in methylation patterns and levels were observed in all sample groups. While these methylation patterns were associated with transcriptional silencing in medulloblastoma cell lines, suggesting that *S100A2* is epigenetically silenced by hypermethylation in the normal cerebellum, no clear evidence of aberrant tumour-specific epigenetic regulation in medulloblastoma development was found, and more detailed examination of extended tumour cohorts are now required to further discern any role in medulloblastoma.

The majority of the *S100* genes, including *S100A2, S100A4, S100A6* and *S100A10* are organised in a cluster on chromosome 1q21.3 (Figure 1). Recent studies have provided precedent for epigenetic silencing across an entire chromosome band in colorectal cancer (Frigola *et al*, 2006); however, this does not appear to be the case for the *S100* gene cluster in medulloblastoma. Microarray analysis of *S100* gene expression changes following demethylation treatment of medulloblastoma cell lines (Figure 1) shows that there is no positional clustering of upregulated genes and the expression of many genes in the cluster remains unchanged. Furthermore, analysis of DNA methylation changes shows that some medulloblastoma cell lines (e.g., MHH-MED1, MEB-MED8A) show both hypermethylation (e.g., of *S100A6*) and hypomethylation (e.g., of *S100A4*) of genes in close physical proximity, suggesting that epigenetic remodelling appears to be localised within the *S100* cluster, involving gene-specific hypermethylation and hypomethylation events. Furthermore, our findings indicate that *S100* gene methylation patterns may have clinical significance and potential utility as medulloblastoma biomarkers. Based on an initial analysis in our limited cohort, methylation of *S100A6* was significantly associated with the more aggressive large cell/anaplastic histopathological variant (Giangaspero *et al*, 2000; McManamy *et al*, 2003), and investigations in large uniformly treated clinical trials cohorts are now required to investigate these relationships further.

A proportion of the CpG methylation found in normal tissue contributes to the silencing of transposable elements and foreign DNA sequences and the cancer genome is known to be globally hypomethylated at these sites, which contributes towards its overall genomic instability (Ehrlich, 2002). However, the role of methylation in the control of expression of individual genes in normal tissues has not been studied in detail and the majority of studies of the methylation status of individual genes in tumours have focused on hypermethylation events leading to the epigenetic inactivation of putative tumour suppressor genes. This has led to the advocation of demethylating agents as anti-cancer therapeutics (reviewed in Strathdee and Brown, 2002). The growing evidence that a number of genes are epigenetically silenced by methylation in normal tissues (reviewed in Strathdee *et al*, 2004) and that some of these genes such as *S100A4* may be tumour promoting, now forces a reconsideration of our understanding of how alterations in the epigenome contribute to cancer development, and the rationale and mechanisms underlying the therapeutic efficacy of such ‘epigenetic’ therapies.

The epigenetic regulation of the *S100* gene family in medulloblastoma and the normal cerebellum illustrates the complex nature of the epigenome, with different family members being subject to both tissue and tumour-specific epigenetic control by DNA methylation, involving both hypomethylation and hypermethylation events which occur during tumourigenesis. Further work is now needed to (i) understand the role of *S100* gene family deregulation in medulloblastoma development and (ii) determine the relative contribution of hypo and hypermethylation events in the development of this tumour and its implications for cancer therapeutics.
